# Integrating traditional Chinese medicine into Chinese medical education reform: issues and challenges

**DOI:** 10.5116/ijme.58e3.c489

**Published:** 2017-04-13

**Authors:** Miao Hua, Jingyi Fan, Hongmei Dong, Renslow Sherer

**Affiliations:** 1 Department of Anthropology, University of Chicago, Chicago, Illinois, USA; 2 Department of Pediatrics, Zhongnan Hospital, Wuhan, China; 3 Department of Medicine, University of Chicago, Chicago, Illinois, USA

**Keywords:** Integrating, traditional Chinese medicine, medical education reform, China

## Introduction

The Chinese state system of medical education has been undergoing reform since the turn of the millennium, with greater impetus since 2008.^1^^,^^2^ These reforms impinge on pluralistic health care institutions. Medical pluralism has thrived over the past 60 years in China within state-run hospitals, universities, and clinics in which biomedicine and traditional Chinese medicine (TCM) are practiced in parallel. By biomedicine, we mean the diagnostic and therapeutic methods and practices first developed in Europe, based on experimentally derived understandings of the body as a biological system. By TCM, we indicate a system of theories and practices based a vast collection of ancient texts that have been enriched with clinical experiences in China and other parts of East Asia for over two millennia, with millions of practitioners in China and across the world today. Despite significant differences between the ways these two medical systems understand the body and treat illnesses, they are often taught and practiced side-by-side within the Chinese state health care system.

Unlike Complementary and Alternative Medicine (CAM) in the US, TCM is a required rather than an optional component of a *biomedical* education in China.^3^ The Chinese Ministry of Health stipulates that undergraduate biomedical universities must provide nationally standardized courses in TCM.^4^ Scholars have previously noted the uniqueness of this pluralistic health care and medical education system.^5^^,^^6^ But issues of limited mutual understanding between TCM and biomedicine persist, so we note the importance of integrating traditional Chinese medicine into medical education reform. In 2013, we surveyed and interviewed over one hundred clinicians and medical students, all trained in biomedicine, at a major provincial teaching hospital in central China. In this paper, we summarize the breadth and the limitations of students and clinicians’ experiences with TCM, and the challenges that they perceive to persist with their TCM education.

### Experiences with TCM among Clinicians and Students of Biomedicine

Given that TCM is already embedded within state health care services, most clinicians and students report basic familiarity with different types of TCM therapeutics, including herbal medicines taken by mouth, methods of physical manipulation on the body surface like *tuina* massage, moxibustion and cupping, and various forms of acupuncture. Our respondents recounted a wide range of conditions from musculoskeletal to digestive for which TCM therapeutics was considered to have some degree of efficacy. A common thread that runs through their rationale for applying TCM to a particular diagnosis is the condition’s chronicity or slowness of onset. This corroborates a commonplace assumption that TCM is effective primarily for treating chronic conditions, for its therapeutic efficacy unfolds slowly, if in due course. Many TCM doctors would contradict this popular assumption, suggesting that it is a misperception that does not consider the efficacy of Chinese medicine in treating acute infectious disorders, for instance.^7^

Such assumptions (or misperceptions) can pigeonhole TCM toward certain types of conditions, such as dysmenorrhea and headaches, while contributing to its uninformed use in others, such as in most forms of cancer therapy. McQuade et al. (2012) found that out of the 77 oncologists they surveyed in Shanghai, 90% admitted to having prescribed some form of TCM treatments.^8^ Most biomedical clinicians we surveyed, including several oncologists, were comfortable only with prescribing Chinese patent medications. The prescription of herbal formula is often deferred to professional TCM doctors. Many clinicians described mixed experience with efficacy, and a lack of systematic understanding of drug interactions.

### Current Limitations of TCM Education

TCM education is a mandatory part of medical education in China. A survey conducted in China over ten years ago shows that students in biomedicine universities receive two semesters of training in traditional Chinese medicine, amounting to over 200 didactic hours.^9^ The clinicians and students we surveyed have almost all received mandatory instructions in TCM, but their opinions were split regarding whether or not such instructions were helpful. Students complained that Chinese medical theory taught in the classroom was challenging and mysterious. Some called for “better integration of theory and practice” in didactics. If given the opportunity to train further in TCM, the majority of students would prefer the clinical setting, citing a desire for more “experiential/practical training”. From this perspective, a movement away from didactics to greater emphasis on case-based learning and problem-based learning in biomedical education in China^10^^,^^11^ may be a welcomed change in TCM education as well.^12^ More important still, clinical rotations in TCM deserve broad implementation at different stages of biomedical training in China to reflect its widespread and cross-specialties clinical applications.

## Conclusions

Medical education in China is in the midst of systematic reform.^13^ Yet, little effort has been made in assessing how biomedical students evaluate their training in TCM, or how issues specific to TCM education bears upon general medical educational reforms. While many clinicians and students trained in biomedicine have used TCM at the bedside, around half of our respondents considered their training in TCM to have been inadequate. Integrating Chinese and Western medicine in education contends with the challenge of bridging significant differences between two medical systems that historically developed apart.  However, their joint use forms an important part of Chinese clinical reality that should be reflected in China’s medical educational reform.

### Conflict of Interest

The authors declare that they have no conflict of interest.
